# Impact of Sucrose Replacement on Physicochemical Properties of Whole-Wheat Biscuits

**DOI:** 10.3390/foods15112032

**Published:** 2026-06-05

**Authors:** Virginia Teresa Glicerina, Hazel Dilşad Tatar, Tawanda Edzai Jeke, Roberta Foligni, Marta Bertolino

**Affiliations:** 1 Department of Agricultural, Forest and Food Sciences, University of Turin, Largo Braccini, 2, 10095 Grugliasco, Italy; 2 Department of Human Science for the Promotion of Quality of Life, San Raffaele Roma University, Via Val Cannuta 247, 00166 Roma, Italy

**Keywords:** polyols, trehalose, rheology, texture, aromatic profile, biscuits

## Abstract

In recent years, consumers have become increasingly aware of the health implications of diets high in sugar. This has prompted the baking industry to engage in the development of low-calorie biscuits while preserving the sensory and technological properties typically provided by sucrose. The present study evaluated the use of five polyols (erythritol, isomalt, maltitol, sorbitol, and xylitol), trehalose, and fructose (included because of its lower postprandial glycemic response compared with sucrose) as sucrose replacers in whole-wheat biscuits by assessing their physicochemical properties and sensory acceptability. The results demonstrated that the isomalt, maltitol and trehalose were able to preserve or improve the structural characteristics of the biscuits. Such an outcome was mainly associated with their ability to promote adequate dough spreadability and to produce biscuits with desirable crispness. Moreover, the biscuits formulated with isomalt and trehalose achieved the highest sensory appreciation, likely due to their high content of 2,4-decadienol, 2-acetatone and 2-pentanone, compounds associated with roasted and buttery flavour notes. Overall, the study demonstrated the feasibility of a complete replacement of sucrose in the production of reduced-calorie biscuits that could be highly acceptable by the consumers, utilizing polyols or low-digestibility sugar such as trehalose.

## 1. Introduction

Biscuits are globally consumed and the increasing demand for healthy and functional foods has encouraged researchers to improve their nutritional quality. One possible strategy is the reduction in the glycemic index through the replacement of sucrose with alternative sweeteners [1,2,3,4,5]. Sucrose plays a key technological role in both dough and final baked products. It attracts and binds water, thereby delaying gluten network formation and starch gelatinization, contributing to the development of a crisp texture. Sucrose also influences colour and aroma development through the browning reactions. Furthermore, by reducing water activity, it slows microbial activity, and contributes to shelf-life extension [6].

For these reasons, both the type and concentration of sucrose replacers are critical factors in bakery product reformulation [6]. Polyols have been extensively proposed as sucrose replacers in bakery products because they provide similar technological functions (acting as a bulking agent). In addition, they provide health benefits because they are only partially digested in the human body, thereby reducing both caloric intake and the glycemic index of the final product. Polyols are sugar alcohols structurally like sugars, in which the aldehyde or ketone group is reduced to a hydroxyl group [7]. They can be classified as monosaccharide polyols (erythritol, mannitol, sorbitol, and xylitol), disaccharide polyols (isomalt and maltitol) and polysaccharide polyols (hydrogenated starch hydrolysates, occurring naturally in many fruits). Polyols are generally recognized as safe and approved by the Food and Drug Administration (FDA). In the bakery industry the most used polyols are xylitol, maltitol, erythritol, sorbitol and isomalt. Xylitol has a sweetness intensity comparable to sucrose while providing approximately 40% less energy. Kutyła-Kupidura et al. [8] underlined that biscuits sweetened with xylitol were only slightly harder than the ones with sucrose and achieved a sensory texture score comparable to the control. Maltitol, which exhibits approximately 90% of the sucrose sweetness, can be used as a near one-to-one sucrose replacer. Biscuits containing maltitol generally show good physical characteristics and high sensory acceptance [9]. Erythritol has approximately 70% of the sweetness of sucrose and is characterized by high digestive tolerance because it causes fewer laxative effects than other polyols. Furthermore, it is considered a non-caloric and non-glycemic sweetener, and has also been associated with antioxidant activity [10]. Erythritol is often combined with maltitol to enhance sweetness perception and moisture retention in baked goods, resulting in compact biscuits with increased elasticity and fracture resistance [11]. Sorbitol exhibits approximately 60% of the sweetness of sucrose and is widely used in the formulation of sugar-free cookies, cakes and muffins [12]. Roze et al. [9] reported that sorbitol-based biscuits exhibited compact structures and were less prone to cracking, although they showed lower hardness than sucrose samples. Isomalt, which possesses approximately 50% of the sucrose sweetness, is characterized by low hygroscopicity and a clean sweet taste without an undesirable aftertaste [13]. The use of isomalt in biscuits has been associated with softer textures and improved consumer acceptability [12].

However, one of the major limitations associated with the use of polyols in foods is their laxative effect when consumed in excessive amounts by the average consumer. Consequently, according to European Regulation (EU) No 1169/2011, Annex III, products containing more than 10% added polyols must bear the advisory statement: “Excessive consumption may produce laxative effects” [14]. Furthermore, polyols may reduce product suitability for individuals affected by irritable bowel syndrome (IBS) and inflammatory bowel disease (IBD), since foods containing fermentable oligo-, di-, monosaccharides and polyols (FODMAPs) may undergo intestinal fermentation and exacerbate gastrointestinal symptoms.

Besides polyols, trehalose, which has been approved as a novel food or food ingredient in Asia, the EU, the UK, and the US has attracted considerable interest due to its unique properties. Trehalose is a non-reducing disaccharide composed of two glucose units, with approximately 45% of the sweetness of sucrose and potential benefits in insulin sensitivity regulation [15]. From a technological perspective, it has a high glass transition temperature and a strong hydrophilicity, and it can provide bulk and texture in the products where it is used [16].

Fructose, a monosaccharide approximately 1.73 times sweeter than sucrose, has also been proposed as a sucrose replacer because of its lower glycemic response [17]. Fructose contributes to several physical and functional properties in food and beverage applications, including sweetness, colour and flavour development, freezing-point depression and osmotic stability [18]. Biscuits produced with fructose are characterized by higher redness value and lower lightness compared to polyol ones because fructose participates in the Maillard reaction. Nevertheless, some polyols may also promote browning owing to their thermal conductivity properties. Furthermore, fructose-containing biscuits are also characterized by higher concentrations of α-dicarbonyl and furanoic compounds, often associated with bitter aftertastes compared with sucrose-based products [19]. However, fructose may also undergo intestinal fermentation, and it is, therefore, classified as a FODMAP ingredient. Consequently, low-FODMAP diets recommend limiting fructose intake to less than 0.15 g per serving when fructose is present in excess relative to glucose [20]. Although several studies have investigated the use of polyols in biscuit formulations, limited information is available regarding their application in whole-wheat biscuits. Moreover, to the best of our knowledge, no previous study has evaluated the application of trehalose in biscuits with a specific focus on physicochemical properties, volatile profile, and sensory characteristics. Therefore, the aim of this study was to evaluate and compare the effect of replacing sucrose with five polyols (erythritol, isomalt, maltitol, sorbitol, and xylitol), the low-digestible disaccharide sugar trehalose and the low-glycemic monosaccharide fructose, on the rheological properties of whole-wheat dough as well as on physicochemical, structural attributes, volatile profile and sensory characteristic of the resulting biscuits.

## 2. Materials and Methods

### 2.1. Ingredients

Erythritol, isomalt, maltitol, sorbitol, trehalose and xylitol were purchased from Sapore Puro (Turin, Italy). The fructose, sucrose, whole-wheat flour (composition expressed as g/100 g of product: carbohydrate 57, protein 13, fibre 12.5, fat 1.8), sunflower oil, and baking powder (disodium diphosphate and sodium carbonate) and water used for the sensory analysis were purchased from a local market.

### 2.2. Biscuit Preparation

The biscuits were prepared according to the formulation reported in Table 1.

A *Classic* KitchenAid^®^ stand mixer 4.3 L (Whirpool Corporation, Benton Harbor, MI, USA) was used to mix the ingredients. The creaming step was carried out by whisking the oil and the sweeteners for 3.5 min at a speed of 7. Water was then added and mixed for an additional one minute. Finally, whole-wheat flour and baking powder were added to the cream and kneaded using a K-hook for 4 min with a speed of 2. After resting for 30 min at 4 °C, the dough was sheeted to a thickness of 3 mm, cut into circular shapes with a diameter of 6 cm and baked in an OLIS oven 044-054S (Ali Group, Milan, Italy) in fan mode for 16 min at 170 °C. After baking, the biscuits were cooled at room temperature for 30 min prior to physicochemical and structural analyses. The biscuits intended for sensory analysis were packaged in an airtight plastic bag and stored at room temperature in dark condition, whereas the samples for GC/MS analysis were milled and stored in an airtight bag at −18 °C until the analysis. Three independent batches were prepared for each formulation.

### 2.3. Rheological and Textural Analyses on Dough

Rheological analyses were performed by employing a controlled stress rheometer (MCR 52, Anton Paar, Graz, Austria). In steady conditions, the analyses were carried out and modelled as described by Liu et al. [21], by increasing the shear rate from 0.01 to 100 1/s and applying the Herschel–Bulkley model to compare samples and better explain results (Equation (1))

(1) $$\tau =\tau_{0}+K\gamma^{n}$$

where τ represents the shear stress (Pa); τ_0_ the yield stress (Pa), expressed as the minimum stress required from the sample to start to flow; *K* the consistency index (Pa⋅s^n^) related to the yield stress; γ denotes the shear rate, and n represents the flow behaviour index, a dimensionless index ranging from 0 to 1.

Yield stress was selected as a representative parameter of viscosity behaviour [22]. However, according with the ISO standard a gap of 1 mm was employed by using plates of 25 mm in diameter [23]. In dynamic conditions the storage (G′) and the loss modulus (G″) values were determined according to the method proposed by Locke et al. [24]. Preliminary strain sweep tests were conducted to identify the linear viscoelastic region (LVR) within a strain range of 0–100%, at a constant frequency of 1 Hz. Frequency sweep tests were subsequently performed at the fixed strain of 1%, increasing the frequency from 1 to 100 Hz. All the samples were analyzed in triplicate.

Dough textural characteristics were evaluated by using a TAXT2i Texture Analyzer^®^ (Stable Micro Systems, Godalming, UK) equipped with a 25 kg cell, and a P/6 probe. Briefly, 90 g of dough was prepared using the dough set (A/DP) to remove the air inside the sample and to obtain a flat surface, and then the analysis was performed, allowing the probe to penetrate the dough to 20 mm at a test speed of 3.00 mm/s with a trigger force of 5 g [25]. The sample firmness was recorded as the maximum positive force value. The analysis was done in duplicate.

### 2.4. Analyses on Biscuits

#### 2.4.1. Physicochemical Analyses

Moisture was measured on 3 g of grinded biscuit, using a MAC210/NH thermo-moisture analyzer (Radwag, Radom, Poland) at 105 °C. Water activity (a_w_) was measured on 2 g of biscuit powder using the water activity analyser PRE AcquaLab (Decagon Devices, Pullman, WA, USA). The CIELAB colour space indices L*, a*, and b* were evaluated on grinded biscuit, using a CM-5 spectrocolorimeter (Konica Minolta, Tokyo, Japan) on transmittance with a measurement area of 8 mm, an angle of observation of 10°, an illuminant D65, a wavelength spectrum between 360 and 740 nm, in Specular Component Excluded mode and the ΔE were evaluated as described by Cantele et al. [26]. All the analyses were done in triplicate.

#### 2.4.2. Structural Analyses

The physical characteristics of the biscuits in terms of width, thickness and spread ratio were evaluated according to the AACC 10-50.05 method [27]. Briefly, six biscuits were arranged horizontally edge-to-edge, and their total width was measured using a ruler. Each biscuit was then rotated by 90°, and the width was measured again. The average width was calculated by dividing the mean value by six and multiplying it by the conversion factor reported in the official method. For thickness determination, six biscuits were stacked vertically, and the total height was recorded. The biscuits were then re-stacked in a random order, and the measurement was repeated. The average thickness was calculated by dividing the mean value by six and multiplying it by the conversion factor specified in the official method.

The spread factor was determined as the ratio between the average width and the average thickness multiplied by the conversion factor reported in the official method. The weight loss and texture analysis were performed according to Rojo-Poveda et al. [28]. The hardness and crispiness of the biscuit were measured using a TAXT2i Texture Analyzer^®^ (Stable Micro Systems, Godalming, UK) equipped with a 25 kg load cell. An HDP-BS cutting blade was used for the analysis at a speed of 1 mm/s and a distance of 3 mm with a trigger force of 5 g. The hardness was expressed as the maximum force needed for breaking the biscuit and the crispness as the number of picks counted in the obtained cutting curve. For each prototype, a set of 12 biscuits was used for measuring these parameters.

#### 2.4.3. Determination of Volatile Profile

The volatile organic compounds (VOCs) were extracted and analyzed by headspace solid phase microextraction–gas chromatography/mass spectrometry (SPME-GC/MS) as reported by Canali et al. [29]. Briefly, 3 g of grinded biscuit was weighed into an extraction vial (10 mL) closed with an aluminum cap containing a PTFE/silicone septum and conditioned at 40 °C for 30 min. Then a 50/30 mm divinylbenzene/carboxen/polydimethylsiloxane (DVB/carboxen/PDMS) fibre (Supelco, Belefonte, PA, USA) was exposed to the headspace of the vial for 10 min at 40 °C using an AOC5000 autosampler (Shimadzu, Tokyo, Japan). Subsequently, the fibre was desorbed in split mode (1:10) at 240 °C for 7 min using helium as the carrier gas (1.5 mL/min). A QP2010 Plus gas chromatograph equipped with a quadrupole mass spectrometer (Shimadzu, Kyoto, Japan) and a Rtx-Wax fused-silica capillary column (30 m × 0.25 mm i.d. × 1.0 mm film tichness) (Phenomenex, Torrance, CA, USA) was used to separate and identify the aroma compounds. The oven temperature was programmed at 40 °C for 10 min, ramped at 3 °C/min to 200 °C, maintained for 3 min and then increased at 10 °C/min to 240 °C, held for 5 min. The detection was performed under electron impact (EI) ionization at 70 eV, by operating in the full-scan acquisition mode with a m/z scanning range from 30 to 250 amu. The ion source and interface temperatures were set at 200 °C and 240 °C. The compounds were identified based on their mass spectra, compared with those in the NIST (National Institute of Standards and Technology) Mass Spectral Library. The total peak area of all the volatiles was assumed as 100% and the relative percentage of the aroma constituents was calculated as % of the total.

#### 2.4.4. Sensory Evaluation

Sensory analysis was performed with a panel of 40 semi-trained panellists (22 females and 18 males of age 20–30 years) to whom the biscuits were given in random order. The panellists were asked to evaluate the biscuits for their appearance, odour, taste, flavour, texture, overall liking on a 9-point hedonic scale (1 = extremely dislike; 9 = remarkably like) and purchase predisposition on a 7-point scale (1 = definitely no; 7 = definitely yes). Water was provided for mouth rinsing between sample tastings. The tests were carried out in an adapted air-conditioned room equipped with white light (6500 °K) at approximately 21 °C.

### 2.5. Statistical Analysis

The results were analyzed by using a one-way analysis of variance (ANOVA) with Duncan’s post hoc test at a 95% confidence level in the SPSS Statistics software (version 29.01, IBM Corp., Armonk, NY, USA). Furthermore, the normalized data of the GC/MS were analyzed by a Principal Component Analysis (PCA) using the *factoextra* package for R software [30]. The Kruskal–Wallis test (test H) (95% confidence level) was used to process the values obtained by the sensory analysis test. The correlation matrices were calculated and visualized using the “corrplot” R package (version 4.5.2).

## 3. Results and Discussion

### 3.1. Rheological and Texture Analyses on Dough

In Table 2 the yield stress (τ_0_) obtained by applying the Herschel model, the elastic (G′) and viscous (G″) moduli and the firmness values are shown. The yield stress of dough samples was strongly influenced by the type of sucrose replacer used. Sucrose-based dough showed the highest yield stress (9326.15 ± 849.42 Pa) compared with the alternative sweeteners (mean value 5793.19 ± 521.54 Pa), underlying a more structured system requiring greater energy to start to flow. Several studies have reported that the molecular weight of sugars is a key factor affecting product viscosity, with disaccharides exerting a greater effect than monosaccharides [31]. In relation to polyols, the observed dough yield stress differences may be explained, as for sugars, due to their molecular characteristics such as weight, spatial structure and the OH groups number [32]. Indeed, maltitol and isomalt, both disaccharide alcohols, produced doughs with higher yield stress value than xylitol and sorbitol ones, which are two monosaccharide alcohols. However, erythritol, despite being a monosaccharide alcohol, showed yield stress not statistically different from fructose and trehalose, having a behaviour more similar to disaccharide sweeteners in terms of yield stress, as previously observed by Laguna et al. [33].

In oscillatory mode, all the dough samples showed G′ higher than the G″ value, indicating predominantly solid-like and elastic behaviour, as typically observed in this kind of product [34]. All the dough samples showed elastic moduli in the range of 105, and even if not significant, differences were highlighted among the samples in relation to this parameter; xylitol dough and sorbitol-containing dough showed a lower G′ parameter coherent with the yield stress trend, in relation to a less elastic and more extensible dough, related to their very low glass transition temperature (Tg) (below room one). As reported by Biliaderis et al. [35], polyols with low Tg values act as plasticizers in the amorphous state, increasing molecular mobility and free volume while reducing intermolecular interactions within the dough matrix. A decrease in Tg promotes the transition from a glass to a rubberier state, reducing elastic modulus and structural rigidity. As a consequence, dough systems containing xylitol and sorbitol exhibited lower resistance to deformation and weaker viscoelastic behaviour, resulting in lower G′ values and higher extensibility [36,37]. Higher G′ values are normally related to a structured dough giving rise to a stiffer dough [38].

Concerning the texture results, no statistical differences were observed. However, xylitol-containing dough and sorbitol-based samples that showed lowest yield stress also presented less dough consistency (mean value 2.98 ± 0.45 N) while the higher value observed in the erythritol samples (5.29 ± 0.68 N) compared to the other ones are in agreement with the results reported by Hao et al. [31]. All the other sweeteners presented intermediate consistency values (mean value 3.97 ± 0.43 N), probably due to the presence of a quite compact dough that exhibited intermediate resistance to stress.

### 3.2. Analyses on Biscuits

#### 3.2.1. Physicochemical Analyses

In Table 3, the moisture, water activity and colorimetric values of the biscuits are reported. Moisture content ranged from 1.20 ± 0.09 to 2.21 ± 0.08%, values lower than those reported in previous studies investigating the use of polyols as sugar substitutes in biscuits [1,7,8,9,12]. This difference may be attributed to the lower dough thickness used during the shaping phase (3 mm) compared with that adopted in other studies (5–7 mm). In general, the biscuits containing polyols exhibited lower moisture content (mean value 1.50 ± 0.16%) than the sucrose-based samples (2.21 ± 0.08%), with the maltitol biscuits showing the lowest value (1.20 ± 0.09%). Polyols containing a higher number of equatorial and exocyclic (OH) groups tend to increase starch gelatinization temperature and reduce water availability, suggesting that polyol stereochemistry plays a significant role in their reactivity [32]. The results obtained from erythritol employment (1.95 ± 0.07%) were comparable to the trehalose ones (1.96 ± 0.11%) maybe in relation to their physicochemical characteristics especially in terms of higher hydrophilicity and low solubility [16,33,39]. The higher moisture observed in sucrose biscuits may be related to its slower and partial dissolution, as well as to sucrose particle size which can contribute to water retention in the biscuit matrix [40]. Water activity (a_w_) values ranged from 0.082 to 0.155 indicating high product stability since all samples remained below the threshold required for the growth of microorganisms or enzymatic activity. The a_w_ of biscuits formulated with erythritol (0.121 ± 0.010) and sorbitol (0.112 ± 0.013) was comparable to that of sucrose-based biscuits (0.115 ± 0.006), whereas xylitol (0.149 ± 0.014) and fructose (0.155 ± 0.021) exhibited considerably higher values and isomalt and maltitol lower values (0.082 ± 0.013 and 0.084 ± 0.013 respectively). A similar trend was reported by Vahid-Dastjerdi et al. [41] who observed comparable patterns in cookies prepared with polyols and sucrose. The study also showed analogues and non-linear correlations with moisture parameters, as evidenced in the present study (Figure S1).

In relation to the colour parameters, the L* value ranged from 46.36 ± 0.97 in the fructose samples to 65.20 ± 0.53 in the maltitol biscuits, while the a* from 6.30 ± 0.05 in the maltitol-based products to 10.94 ± 0.29 in the erythritol samples, and the b* from 22.31 ± 0.27 in the maltitol biscuits to 31.93 ± 0.44 in the fructose-containing products. The fructose biscuits exhibited the lowest lightness (L*) values, indicating a darker colour (Figure S2). This behaviour is likely attributable to the reducing nature of fructose, which readily participates in the Maillard reaction and promotes the formation of darker products, as also reflected by the higher redness index (a*) values and by the correlation between the two parameters (r^2^ = −0.94; *p* < 0.001) (Figure S1). Instead, polyols and trehalose, owing to the absence of aldo and keto groups, do not participate in the Maillard reaction, resulting in lighter products with lower redness values than fructose-containing biscuits. Sucrose can instead produce browning effects being involved in the Maillard reaction if its thermal degradation and hydrolyzation into glucose and fructose occur [42]. In our study, the biscuits formulated with polyol-based sweeteners showed higher lightness and lower redness (mean score 54.89 ± 1.04 and 9.12 ± 0.28 respectively) than the fructose-containing samples (46.36 ± 0.97 and 13.92 ± 0.15 respectively). However, compared with the sucrose-based biscuits (60.13 ± 3.63 and 7.87 ± 0.43 respectively), they generally exhibited lower lightness and higher redness values, with the exception of the maltitol samples (65.20 ± 0.53 and 6.30 ± 0.05 respectively), which showed values comparable to the control (sucrose). Similar trends were reported by Roze et al. [9] and Psimoulim & Oreopoulou [43]. The colour differences among the samples may be related to the different physicochemical properties of the polyols, in terms of hygroscopicity, solubility, and glass transition behaviour, which can affect water mobility and moisture distribution during baking. In particular, the slower migration of water through the biscuit surface may promote localized dehydration and thermally induced browning phenomena [6,7,11]. Therefore, in our study the higher red index (a*) and lower lightness (L*) showed by erythritol, sorbitol, isomalt and xylitol biscuits with respect to sucrose can be reasonably attributed to a partial degradation of sucrose and to the lower migration of the water through the surface in the polyol-based biscuits. Moreover the trehalose biscuits presented lightness values like the maltitol ones. For b* value, significant differences were noted among the samples and increasing the a* value also results in a higher b*, in relation to the positive correlation among the two parameters (r^2^ = 0.90; *p* < 0.001 (Figure S1). Regarding the ΔE values, all the samples showed differences higher than 2.5 when assessing their colour in comparison to the control (sucrose), with the lowest value in the trehalose biscuits (3.61 ± 1.01) and the highest in fructose (16.34 ± 2.15) meaning that the sugar substitution can be easily perceived by the human eye and therefore accepted or not by the consumers.

#### 3.2.2. Structural Analyses

In Table 4 the physical parameters (weight loss, width, thickness, spread ratio) and the textural attributes (hardness and crispiness) of the biscuits are shown. Misra et al. [37] and Rojo-Poveda et al. [28] reported that ingredient characteristics, particularly the solubility and water-binding capacity of sugars and polyols, strongly influence the physical properties of products.

The width of the biscuits ranged from 4.59 ± 0.08 to 4.96 ± 0.06 cm. The biscuits formulated with sorbitol, which also showed the lowest dough yield stress, and maltitol were found to be the largest (4.96 ± 0.06 and 4.91 ± 0.04 cm respectively). This behaviour may be attributed to their higher solubility compared with sucrose, which promotes dough flow as reported by Roze et al. [9] and according to Zoulias et al. [5] to their influence on dough setting time which affects both gluten network and glass transition properties. Instead, the biscuits produced with erythritol and fructose showed the lowest width (4.65 ± 0.02 and 4.59 ± 0.08 cm respectively) and higher thickness values (0.60 ± 0.03 and 0.70 ± 0.02 cm respectively), likely because of lower solubility and higher dough yield stress, which generated stiffer dough systems prone to shrinkage during baking as observed in confectionary products [44]. Moreover, in relation to thickness a positive correlation has been highlighted between its decrease and viscosity values in terms of yield stress (r^2^ = 0.75; *p* < 0.001) (Figure S1) as previously reported by Ho and Pulsawat [12]. Spread ratio represents an index able to evaluate the influence of sucrose and its replacers on both the width and thickness of biscuits into a single parameter. It is considered one of the main quality indicators in biscuit production because it affects processing efficiency, especially in the automated manufacturing, as well as texture, bite and overall mouthfeel of the final products [45]. Also, for this parameter, an inverse correlation trend with dough viscosity has been highlighted for all samples (r^2^ = −0.79; *p* < 0.001) (Figure S1). According to Zoulias et al. [5], the spread of cookies is controlled by the rate at which the dough flows, and this one is associated with the viscosity of the prepared dough. The polyol-based (mean value 74.04 ± 2.46) and trehalose-based biscuits (85.31 ± 2.11) showed better spreading than the sucrose and fructose ones (72.71 ± 1.62 and 64.96 ± 2.52 respectively) with the sorbitol ones characterized by the highest spread ratio (103.98 ± 1.09).

The biscuits produced with maltitol, isomalt, and trehalose exhibited an average hardness reduced by 29% (mean value 58.20 ± 17.76 N) compared with the sucrose-containing samples (81.59 ± 22.51 N). For maltitol the formation of smaller crystals with respect to sucrose size during cooling probably contributed to a softer texture as reported by BeMiller [46]. A similar behaviour could be reasonably attributed to isomalt which, according to Mosafa et al. [47], showed a softening effect in biscuits. Concerning trehalose, a softening effect on cookies was also observed by Kawai et al. [48], in relation to its high glass transition and water sorption properties. Conversely, the erythritol- and fructose-based biscuits showed a marked increase in hardness (116.24 ± 40.21 and 105.37 ± 38.30 N respectively), approximately 172% higher than sucrose-formulated samples. This behaviour is likely associated with differences in water interactions and solubility, as previously stated. This, in turn, gives rise to different structures (higher viscosity and elasticity) and resistance to breakage [12]. Similar outcomes were obtained by Laguna et al. [33], wherein erythritol 50% biscuits exhibited a notably higher fracture force compared to sucrose at equivalent concentration. Concerning fructose results, the explanation can also be associated with its partial thermodynamic dissolution or re-crystallization in larger crystals as observed by Zoulias et al. [5].

Regarding crunchiness, the polyol biscuits were generally less crispy (mean value 24.16 ± 15.60 picks) than the sugar-based biscuits (mean value 39.06 ± 21.17 picks), in relation to their higher humectant effect with respect to sugars, but also due to their lower crystallization rate and lower Tg, leading to less fracturable products [12,49]. Furthermore, erythritol biscuits, besides showing the highest hardness value (116.24 ± 40.21 N), also exhibited the lowest friability (15.75 ± 11.76 picks), probably related to the low hygroscopicity of the substitute, which allowed the samples to dry faster, favouring the formation of biscuits with a lower porosity [31]. The biscuits containing sucrose, fructose and trehalose showed similar crispness values, not statistically different among them, probably in relation to their small-pore-size inner structure [40].

#### 3.2.3. Volatile Organic Compounds Analysis

The volatile organic compounds identified and their relative contribution to the total volatile profile of the biscuits are presented in Table S1. Overall, 51 volatile molecules were identified, including 10 aldehydes, eight alcohols, eight carboxylic acids, eight ketones, seven esters, six furan compounds, three pyrazines and one terpene. Ketones and aldehydes represented the predominant volatile classes, accounting for 30% and 28% (mean value among samples) of the total volatile compounds, respectively. The main representative ketone in all samples was the 2-heptanone followed by acetone in the polyol-based biscuits and 4-hydroxy-4-methyl-2-pentanone in the other biscuits in line with Rutkowska et al. [50].

Ketones are mainly generated through the lipid oxidation of unsaturated fats under thermal conditions, as well as through enzymatic hydrolysis processes due to endogenous lipases of the flour, which promote the hydrolysis of triglycerides. The resulting free fatty acids, under baking temperatures, can subsequently lead to the formation of ketones [51,52,53]. The higher concentration of acetone in the polyol-based biscuits could be due to a lower antioxidant effect of sugar alcohols compared to the mono- and disaccharides [54]. Trehalose, being a sucrose isomer, can show the same scavenging behaviour, as reported in Darikvand et al. [55]. Related to fructose biscuits, their lower acetone concentration can be probably attributed to the intense Maillard reaction participation of fructose and to its end-products (melanoidins) that according to Nooshkam et al. [56] are characterized by strong antioxidant activity.

The preponderance of the detected aldehydes was in line with previous findings related to biscuits, with 2-methylbutanal, 3-methylbutanal, hexanal and nonanal being the most abundant ones. The first two are produced by Strecker degradation processes of the amino acids isoleucine and leucine respectively, and they contribute to the pleasant malty odour of biscuits [57,58]. On the contrary, hexanal and nonanal derived from the auto-oxidation of linoleic acid [58] are related to the sensory attribute of rancidity, as observed in biscuits by Balestra et al. [59].

Among alcohols, ethanol was the most abundant and its presence in bakery products is normally associated with yeast fermentation. However, in the absence of biological leavening, as in the present work, its detection could be associated with unpredictable microbiologic activities occurring during the wheat grain storage [60] or during the production process [61]. Moreover, ethanol may also originate from thermal degradation pathways occurring during baking including Maillard reactions, the caramelization of sugars, lipid oxidation, and Strecker degradation reactions, which can generate acetaldehyde subsequently converted into ethanol [62].

Acetic acid, generated through Maillard reactions and sugar caramelization, was the predominant carboxylic acid detected in biscuits, and it reflected the amount of ethanol recorded. In their studies, Cincotta et al. [19] identified acetic acid as a common volatile compound present in biscuits containing sucrose, fructose, or glucose, and Rutkowska et al. [50] found it in biscuits containing xylitol.

Ethyl acetate, the ester of acetic acid, was detected in polyol-based biscuits, but was absent in sucrose and fructose and present at a lower concentration in the trehalose ones. Although ester formation in baked products is frequently associated with yeast fermentation, such compounds have also been identified in biscuits produced without yeast addition [63] and in formulations containing sugar alcohols such as xylitol [50]. In the present study, a plausible explanation for the detection of esters may be related to the natural occurrence of volatile compounds in wheat flour, which could represent an intrinsic source, in agreement with Pico et al. [64].

Among Maillard reaction markers, furan compounds were more abundant in the sugar-based biscuits, particularly those containing fructose, whereas pyrazines predominated in the polyol-based samples. Regarding fructose, our findings corroborate the study of Srivastava et al. [65] which stated that the presence of reducing sugars leads to greater levels of furfural. Moreover, in agreement with Guo et al. [42] and Mesías et al. [66] the use of polyols, which inhibited the Maillard reaction, led to a decrease in furan compounds, representing a valid strategy to mitigate the formation of these compounds. Although furan compounds might be concerning because of their possible carcinogenicity, the presence of pyrazines is desirable because these compounds contribute to roasted aroma notes. Methylpyrazine and 2,5-dimethylpyrazine were the most abundant compounds detected, and these results are in common with the ones of Rutkowska et al. [52] where xylitol was used as a sucrose substitute.

The terpene d-limonene was also detected in all the samples, and may derive from carotenoid degradation in the sunflower oil [67,68] or could be a natural compound present in the wheat flour as already reported by Pico et al. [64].

GC-MS data were further analyzed through Principal Component Analysis (PCA) to highlight differences among the samples and evaluate the impact of sweeteners used in the formulation on the volatile profile of the final products.

**Figure 1:**
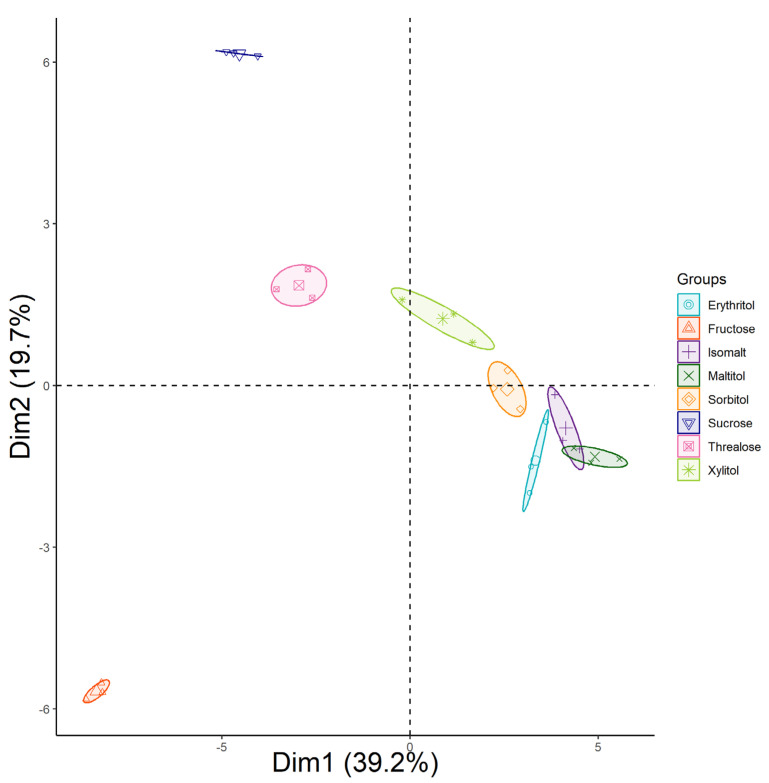
A score plot of the first two PCA dimensions obtained from the GC/MS data of biscuits.

As showed in Figure 1, the first two principal components collectively explained 58.9% of the total variance. A clear separation among the samples was observed. The first dimension accounted for 39.2% and was able to differentiate the sugars that exhibited negative values from the polyols that exhibited positive values. The compounds that allowed us to separate the sugars were the furan compounds and particularly the furanesol and 5-Hydroxymethyl furfural (HMF) while the polyols were correlated with the aldehydes and especially with the pentanal and benzaldehyde (Figure S3). The second dimension accounted for 19.7% of the variance and allowed us to separate the disaccharides (sucrose and trehalose) from the monosaccharide (fructose) thanks to the butanoic acid and the 2,5-dimethylpyrazine concentration. Among the polyols only xylitol was clearly separated, exhibiting a positive score value along dimension 2 while all the others were characterized by negative values.

#### 3.2.4. Sensory Analysis

In Table 5 the results of the sensory analysis are reported as the sum of ranks calculated for each biscuit. Colour is one of the primary sensory attributes influencing consumer preference and product selection. Fructose-containing biscuits were the least appreciated (mean score of 3.60 ± 2.16; Table S2) because of their dark and reddish appearance, which resembled overcooked or burnt product, as previously stated in the colorimetric results part, while no statistical differences were observed among the other samples (mean score 6.22 ± 1.67). The extensive Maillard reaction promoted by fructose also negatively affects aroma perception (mean score 2.00 ± 0.96) because of the bitter taste produced by furan compounds, especially furfuryl alcohol generated during the reaction as observed in the biscuits’ volatile profile and as underlined by Starowicz et al. [69].

To better evaluate the taste performance of the obtained products, the panellists were asked to evaluate their sweetness. Once again, the fructose-containing biscuits achieved the lowest score (2.60 ± 1.77) while among the polyols used the most appreciated was the xylitol one (5.57 ± 1.46) that obtained the same score as the sucrose (5.55 ± 2.21) from a statistical point of view. From the correlation analysis, sweet taste was found to be strongly influenced (*p* < 0.001) by the content of carboxylic acids (r^2^ = 0.66), followed by ketones (r^2^ = 0.52; *p* < 0.01), whereas furan compounds showed a negative correlation with sweetness perception (r^2^ = −0.48; *p* < 0.05) (Figure S4). Indeed, the fructose-based biscuits were characterized by the lowest concentration of carboxylic acid and the highest concentration of furan compounds. Among the polyols, the higher appreciation of sweet taste can be attributed to the relatively high sweetness of xylitol, which is comparable to that of sucrose. Regarding flavour, the isomalt- and trehalose-based samples were highly appreciated and perceived as the most aromatic biscuits even if not statistically different from sucrose and erythritol, probably in relation to the presence of 2,4-decadienal in a higher amount, which is normally related to the aroma of a roasty nut flavour [70]. In addition, linear ketones such as 2-pentanone and 2-heptanone are normally perceived with fruity and pleasant characteristics that resemble cooked butter [57,71] and were detected in high concentration in the isomalt and trehalose biscuits respectively. Regarding structural attributes, the samples formulated with erythritol, followed by the fructose samples, received the lowest texture appreciation scores (3.40 ± 1.88 and 5.15 ± 2.65 respectively), in agreement with the previous textural analysis that highlighted a very high hardness for these two biscuits, resembling a rigid compact structure that is difficult to break with teeth. For overall liking, the xylitol- and isomalt-based biscuits achieved the highest appreciation scores (5.80 ± 1.49 and 5.75 ± 1.15 respectively). Isomalt was preferred because of its soft texture (as confirmed by texture analysis), while xylitol was appreciated mainly for its sweet taste. Specifically, according to Erdem et al. [72], sweetness perception seems to be related to the presence of three volatile compounds: 2-methylbutanal and 3-methylbutanal, responsible for a malt or cocoa flavour; and phenylacetaldehyde, responsible for a floral and honey-like aroma. In the xylitol-based biscuits, these volatiles were presented in higher amounts compared to the other polyols, while the sorbitol ones showed the lowest amounts; in addition, phenylacetaldehyde was detected only in the xylitol and sucrose biscuits. These perceptions influenced also the willingness to buy; in fact, isomalt received a positive answer by the panellists followed by trehalose and xylitol. In the case of trehalose, the texture, flavour and taste influenced the judgement.

## 4. Conclusions

This study has demonstrated that the complete replacement of sucrose in whole-wheat biscuits is technologically feasible. Moreover, the study emphasized the correlation between the molecular and functional characteristics of the sweetener and the rheology of the dough, the structure of the biscuit, its flavour profile and its sensory perception.

Among the sweeteners tested, isomalt and trehalose were identified as the most promising alternatives due to their proven ability to preserve the desired structural and sensory characteristics, including spreadability, crispness, flavour development and sensory acceptability. Furthermore, xylitol was highly rated by the panel, mainly for its perceived sweetness and flavour profile.

From an industrial perspective, these results may provide a scientific basis for the development of whole-wheat biscuits with reduced sucrose content that offer improved sensory quality and a reduced glycemic impact. However, a rigorous evaluation of the practicality of polyols and alternative sugars is required, taking into account their recognized limitations. It has been demonstrated that excessive intake of polyols can induce laxative effects; furthermore, their consumption may be contraindicated in cases of gastrointestinal disorders, including irritable bowel syndrome (IBS) and inflammatory bowel disease (IBD). Moreover, disparities in hygroscopicity, solubility, crystallization behaviour and thermal conductivity have the potential to generate technological challenges during processing, thereby impacting texture stability and consumer acceptance.

Consequently, the selection of sucrose substitutes should not be based solely on calorie reduction or glycemic response, but also on technological performance, sensory quality, digestive tolerance and consumer perception. Therefore, a possible future development of this work could involve the study of combinations of different polyols, as well as combinations of polyols and trehalose, in order to obtain biscuit formulations that more faithfully replicate the technological behaviour of sucrose, whilst achieving a comparable degree of sweetness and mitigating the individual disadvantages of polyols and fructose. Such approaches have the potential to address the functional limitations of individual sweeteners, thereby enhancing the overall quality and consumer acceptability of wholemeal biscuits with reduced sucrose content.

## Figures and Tables

**Table 1:** Formulation of the eight biscuits produced with different polyols and sugars.

Ingredients (g)	Erythritol	Isomalt	Maltitol	Sorbitol	Xylitol	Fructose	Trhealose	Sucrose	Value %
**Whole-Wheat flour**	156.7	156.7	156.7	156.7	156.7	156.7	156.7	156.7	58.3%
**Sugar**	-	-	-	-	-	30	30	30	11.2%
**Polyol**	30	30	30	30	30	-	-	-	
**Baking Powder**	2.7	2.7	2.7	2.7	2.7	2.7	2.7	2.7	1.0%
**Sunflower Oil**	30	30	30	30	30	30	30	30	11.2%
**Water**	49	49	49	49	49	49	49	49	18.3%

**Table 2:** Results (means ± standard deviation) of yield stress (τ_0_), elastic (G′) and viscous (G″) moduli and firmness of dough samples obtained by fundamental rheological and textural analyses.

Samples	τ_0_ (Pa)	G′ (Pa)	G″ (Pa)	Firmness (N)
Erythritol	6977.20 ± 288.07 ^b^	2.31 × 10^5^ ± 2,212.13	8.16 × 10^4^ ± 2699.73 ^bcd^	5.29 ± 0.68
Isomalt	5533.50 ± 956.03 ^c^	1.36 × 10^5^ ± 30,485.63	1.27 × 10^4^ ± 15,736.15 ^ab^	4.57 ± 1.47
Maltitol	5822.13 ± 846.13 ^c^	1.78 × 10^5^ ± 20,896.04	1.11 × 10^4^ ± 18,800.04 ^abc^	4.27 ± 0.15
Sorbitol	3820.55 ± 251.94 ^d^	1.01 × 10^5^ ± 4188	5.89 × 10^4^ ± 8327.39 ^d^	3.15 ± 0.69
Xylitol	4272.75 ± 227.63 ^d^	1.15 × 10^5^ ± 9916.18	6.60 × 10^4^ ± 6311.82 ^cd^	2.80 ± 0.20
Fructose	7091.70 ± 204.07 ^b^	1.48 × 10^5^ ± 23,434.42	8.40 × 10^4^ ± 11,293.64 ^bcd^	3.67 ± 0.06
Trehalose	7034.50 ± 876.93 ^b^	1.57 × 10^5^ ± 118,169	1.49 × 10^4^ ± 60,028.42 ^a^	3.58 ± 0.04
Sucrose	9326.15 ± 849.42 ^a^	1.69 × 10^5^ ± 22,450.64	8.95 × 10^4^ ± 23,218.56 ^bcd^	3.79 ± 0.45
*Sig.*	***	*n.s.*	**	*n.s.*

Results of ANOVA with Duncans’s post hoc test are reported between samples; values followed by different letters are significantly different at *p* < 0.05. *Sig.*, statistical significance; the asterisks denote the level of significance: ** *p* < 0.01; *** *p* < 0.001.

**Table 3:** The results (means ± standard deviation) of moisture, water activity (a_w_), and CIELAB parameters (L* lightness, a* redness and b* yellowness) and the difference among two colours (sucrose and studied sugars/polyols) (∆E) of the biscuits.

Samples	Moisture (%)	a_w_	L*	a*	b*	ΔE
Erythritol	1.95 ± 0.07 ^ab^	0.121 ± 0.010 ^b^	50.15 ± 0.99 ^d^	10.94 ± 0.29 ^b^	27.34 ± 0.12 ^b^	10.59 ± 2.15 ^b^
Isomalt	1.45 ± 0.07 ^cd^	0.082 ± 0.013 ^c^	55.97 ± 1.89 ^c^	8.51 ± 0.49 ^d^	25.97 ± 1.10 ^cd^	4.23 ± 1.02 ^cd^
Maltitol	1.20 ± 0.09 ^d^	0.084 ± 0.013 ^c^	65.20 ± 0.53 ^a^	6.30 ± 0.05 ^f^	22.31 ± 0.27 ^e^	6.20 ± 2.08 ^c^
Sorbitol	1.37 ± 0.21 ^cd^	0.112 ± 0.013 ^b^	51.86 ± 0.81 ^d^	9.72 ± 0.13 ^c^	25.10 ± 0.38 ^d^	8.48 ± 1.44 ^c^
Xylitol	1.54 ± 0.34 ^cd^	0.149 ± 0.014 ^a^	51.29 ± 0.96 ^d^	10.12 ± 0.46 ^c^	26.39 ± 1.05 ^bc^	9.16 ± 1.83 ^c^
Fructose	1.71 ± 0.35 ^bc^	0.155 ± 0.021 ^a^	46.36 ± 0.97 ^e^	13.92 ± 0.15 ^a^	31.93 ± 0.44 ^a^	16.34 ± 1.26 ^a^
Trehalose	1.96 ± 0.11 ^ab^	0.077 ± 0.009 ^c^	63.54 ± 0.42 ^a^	6.71 ± 0.29 ^f^	25.20 ± 0.66 ^d^	3.61 ± 1.01 ^d^
Sucrose	2.21 ± 0.08 ^a^	0.115 ± 0.006 ^b^	60.13 ± 3.63 ^b^	7.87 ± 0.43 ^e^	25.52 ± 0.15 ^cd^	-
*Sig.*	***	***	***	***	***	

Results of ANOVA with Duncans’s post hoc test are reported between samples; values followed by different letters are significantly different at *p* < 0.05. *Sig.*, statistical significance; the asterisks denote the level of significance: *** *p* < 0.001.

**Table 4:** Results (means ± standard deviation) of physical (weight loss, width, thickness, spread ratio) and textural (hardness and crispiness) parameters of different biscuits containing sucrose and different replacers.

Samples	Weight Loss (%)	Width (cm)	Thickness (cm)	Spread Ratio	Hardness (N)	Crispiness (Number of Picks)
Erythritol	2.06 ± 0.01 ^bc^	4.65 ± 0.02 ^c^	0.60 ± 0.03 ^c^	76.46 ± 3.47 ^d^	116.24 ± 40.21 ^a^	15.75 ± 11.76 ^d^
Isomalt	2.00 ± 0.02 ^cd^	4.83 ± 0.05 ^b^	0.52 ± 0.01 ^e^	92.58 ± 1.87 ^b^	56.71 ± 20.47 ^c^	31.05 ± 16.54 ^abc^
Maltitol	2.07 ± 0.04 ^bc^	4.91 ± 0.04 ^a^	0.56 ± 0.01 ^d^	87.49 ± 2.36 ^c^	60.05 ± 16.14 ^c^	32.87 ± 19.65 ^ab^
Sorbitol	1.97 ± 0.00 ^d^	4.96 ± 0.06 ^a^	0.47 ± 0.01 ^f^	103.98 ± 1.09 ^a^	88.10 ± 20.14 ^b^	21.65 ±14.05 ^bcd^
Xylitol	1.96 ± 0.03 ^d^	4.82 ± 0.04 ^b^	0.52 ± 0.02 ^e^	92.71 ± 3.50 ^b^	74.73 ± 31.97 ^bc^	19.50 ± 16.02 ^cd^
Fructose	2.10 ± 0.07 ^b^	4.59 ± 0.08 ^c^	0.70 ± 0.02 ^a^	64.96 ± 2.52 ^f^	105.37 ± 38.30 ^a^	36.77 ± 27.76 ^a^
Trehalose	2.08 ± 0.03 ^bc^	4.81 ± 0.07 ^b^	0.56 ± 0.01 ^d^	85.31 ± 2.11 ^c^	57.84 ± 16.68 ^c^	40.25 ± 15.81 ^a^
Sucrose	2.21 ± 0.00 ^a^	4.76 ± 0.04 ^b^	0.65 ± 0.02 ^b^	72.71 ± 1.62 ^e^	81.59 ± 22.51 ^b^	40.15 ± 19.93 ^a^
*Sig.*	***	***	***	***	***	***

Results of ANOVA with Duncans’s post hoc test are reported between samples; values followed by different letters are significantly different at *p* < 0.05. *Sig.*, statistical significance; the asterisks denote the level of significance: *** *p* < 0.001.

**Table 5:** The results of the Kruskal–Wallis test on sensory analysis evaluation. The data are expressed as the sum of ranks.

	Erythritol	Isomalt	Maltitol	Sorbitol	Xylitol	Fructose	Trehalose	Sucrose	*Sig.*
**Colour**	6534 ^a^	7854 ^a^	6952 ^a^	7358 ^a^	6406 ^a^	2882 ^b^	6464 ^a^	6910 ^a^	***
**Taste**	5002 ^d^	8068 ^ab^	6638 ^bc^	5292 ^cd^	8668 ^ab^	1480 ^f^	7256 ^ab^	8956 ^a^	***
**Sweetness**	5586 ^c^	6410 ^bc^	6258 ^bc^	5690 ^c^	8670 ^a^	3232 ^d^	7490 ^abc^	8024 ^ab^	***
**Flavour**	7244 ^a^	8002 ^a^	6519 ^ab^	4515 ^b^	6716 ^a^	2769 ^c^	7963 ^a^	7632 ^a^	***
**Texture**	2659 ^c^	7715 ^a^	7495 ^a^	6554 ^ab^	6495 ^ab^	5682 ^b^	7648 ^a^	7112 ^ab^	***
**Overall**	4506 ^d^	8170 ^ab^	6578 ^bc^	5330 ^cd^	8216 ^ab^	2476 ^e^	7118 ^abc^	8966 ^a^	***
**Purchase Interest**	5092 ^d^	7496 ^abc^	6488 ^bcd^	5398 ^cd^	9102 ^a^	2660 ^e^	7152 ^abc^	7972 ^ab^	***

Values followed by different letters are significantly different at *p* < 0.05. *Sig.*, statistical significance; the asterisks denote the level of significance: *** *p* < 0.001.

## Data Availability

The original contributions presented in this study are included in the article/Supplementary Material. Further inquiries can be directed to the corresponding author.

## Supplementary Materials

The following supporting information can be downloaded at https://www.mdpi.com/article/10.3390/foods15112032/s1, Figure S1: Pearson correlation matrix among the physicochemical analyses of the biscuits; Figure S2: Visual comparison between the different biscuits; Figure S3: Contribution plot of the volatile compounds to the PCA; Figure S4: Pearson correlation matrix between the identified volatile compounds (expressed as sums) and the sensory analysis scores of the biscuits; Table S1: Volatile compounds detected in biscuits produced with sucrose and different replacers; Table S2: Average values obtained for the biscuits during sensory analysis.
