# Supplementary material for: Impact of Sucrose Replacement on Physicochemical Properties of Whole-Wheat Biscuits
Source: Foods. 2026 Jun 5;15(11):2032. doi: 10.3390/foods15112032 (PMC13256550; doi:10.3390/foods15112032)
Supplement: Supplementary file 1 [file foods-15-02032-s001.zip › Figure S1.pdf]

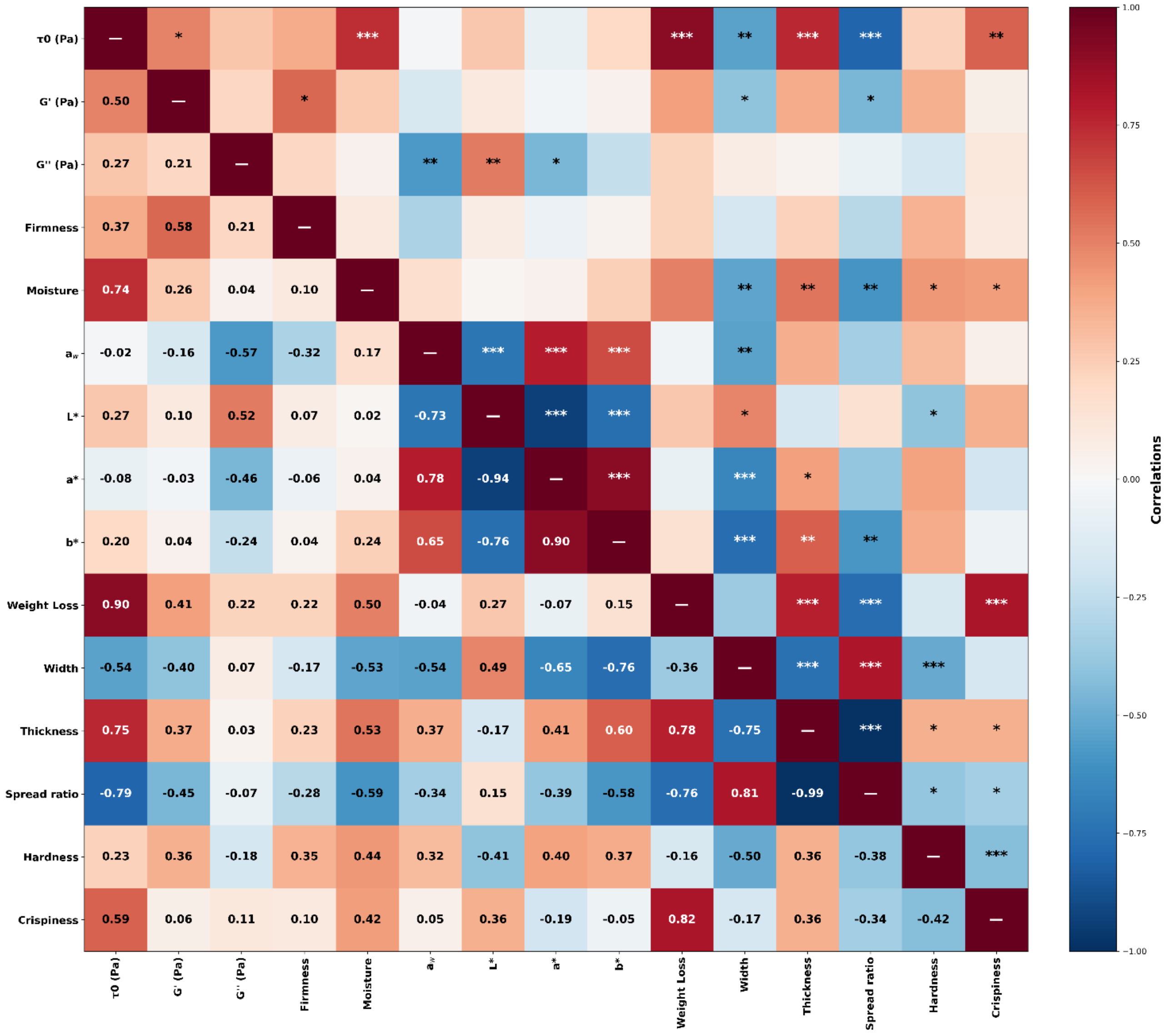

Figure S1. Pearson correlation matrix among the physicochemical analyses of the biscuit samples.

Colour intensity indicates the strength of the correlation. The statical significance of the correlation is reported in the upper/right part of the figure (where * = $p < 0.05$; ** = $p<0.01$; *** = $p<0.001$).
