# Supplementary material for: Impact of Sucrose Replacement on Physicochemical Properties of Whole-Wheat Biscuits
Source: Foods. 2026 Jun 5;15(11):2032. doi: 10.3390/foods15112032 (PMC13256550; doi:10.3390/foods15112032)
Supplement: Supplementary file 1 [file foods-15-02032-s001.zip › Figure S3.pdf]

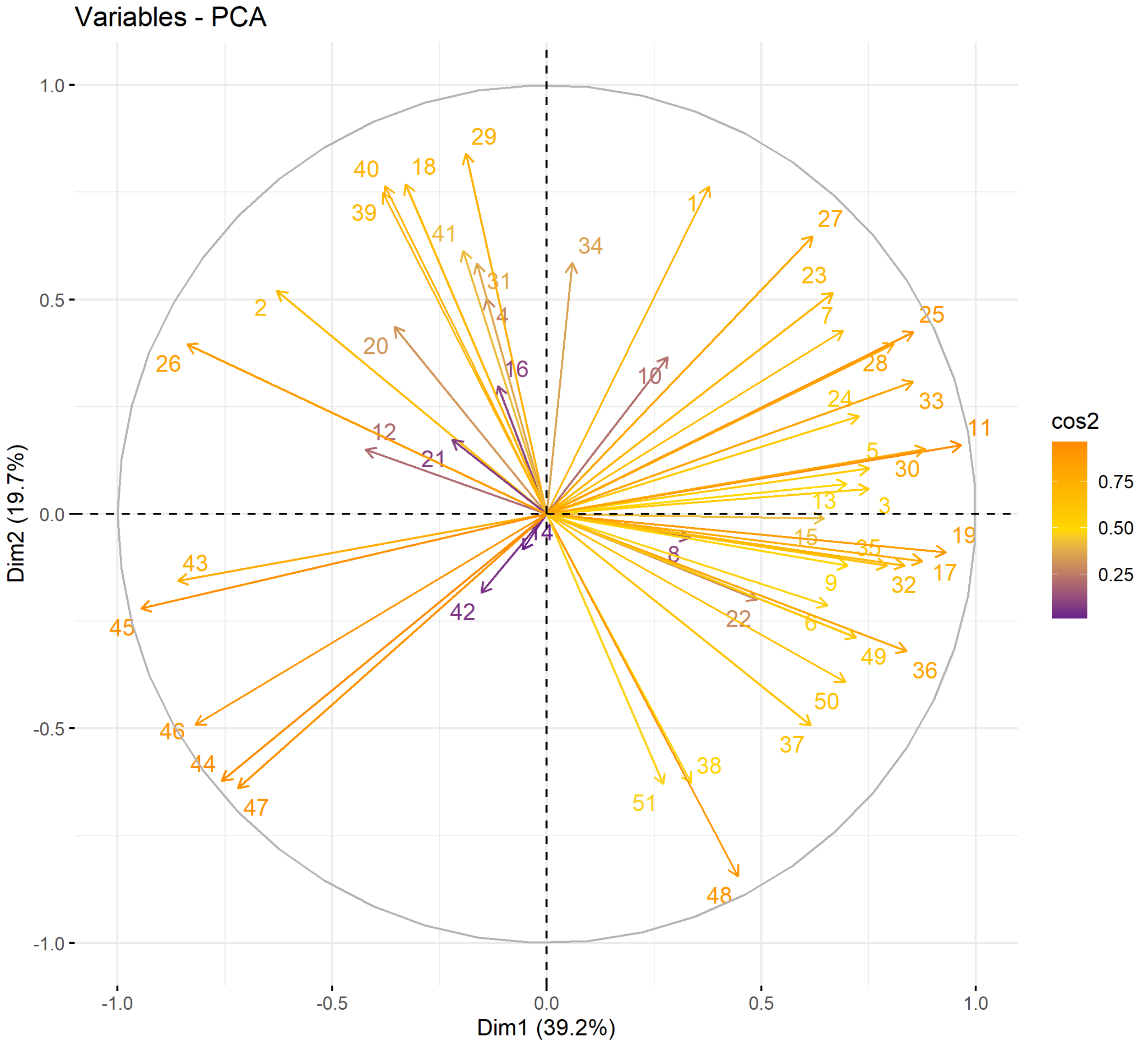

Figure S3. Contribution plot of the volatile compounds to the PCA analysis

1: Ethanol; **2**: 1-Pentanol; **3**: 1-Hexanol; **4**: Hexanol; **5**:Heptanol; **6**: 1-Octanol; **7**: 1-Octen-3-ol; **8**: Benzyl alcohol; **9**: 2-Methylbutanal; **10**: 3-Methylbutanal; **11**: Pentanal; **12**: 2-pentanal; **13**: Hexanal; **14**: Heptanal; **15**: Nonanal; **16**: (E,E)-2,4-Decadienal; **17**: Benzaldehyde; **18**: Phenylacetaldehyde; **19**: Acetone; **20**: 3-Hydroxy-2- Butanone (Acetoin); **21**: 2-Pentanone; **22**: 4-cyclopentene-1,3 dione; **23**: 2-Heptanone; **24**: 2-Nonanone; **25**: 2-Undecanone; **26**: 4-Hydroxy-4-methyl-2-pentanone; **27**: Acetic acid; **28**: Propanoic acid; **29**: Butanoic acid; **30**: Hexanoic acid, **31**: Octanoic acid; **32**: 2-Hexenoic acid, **33**: Nonanoic acid; **34**: Decanoic acid; **35**: Ethyl acetate; **36**: Ethyl butyrate; **37**: Butyl acetate; **38**: Butyl 3-methylbutyrate; **39**: Ethyl hexanoate; **40**: Methyl benzoate; **41**: Ethyl benzoate; **42**: 2-Pentylfuran; **43**: 2-Furanmethanol, **44**: Furfural, **45**: Furaneol; **46**: 5-Hydroxymethylfurfural (HMF); **47**: Furfuryl alcohol; **48**: 2,5-Dimethylpyrazine; **49**: Methylpyrazine; **50**: 2-Methylpyrazine; **51**: d-Limonene.
