# Supplementary material for: Impact of Sucrose Replacement on Physicochemical Properties of Whole-Wheat Biscuits
Source: Foods. 2026 Jun 5;15(11):2032. doi: 10.3390/foods15112032 (PMC13256550; doi:10.3390/foods15112032)
Supplement: Supplementary file 1 [file foods-15-02032-s001.zip › Figure S4.pdf]

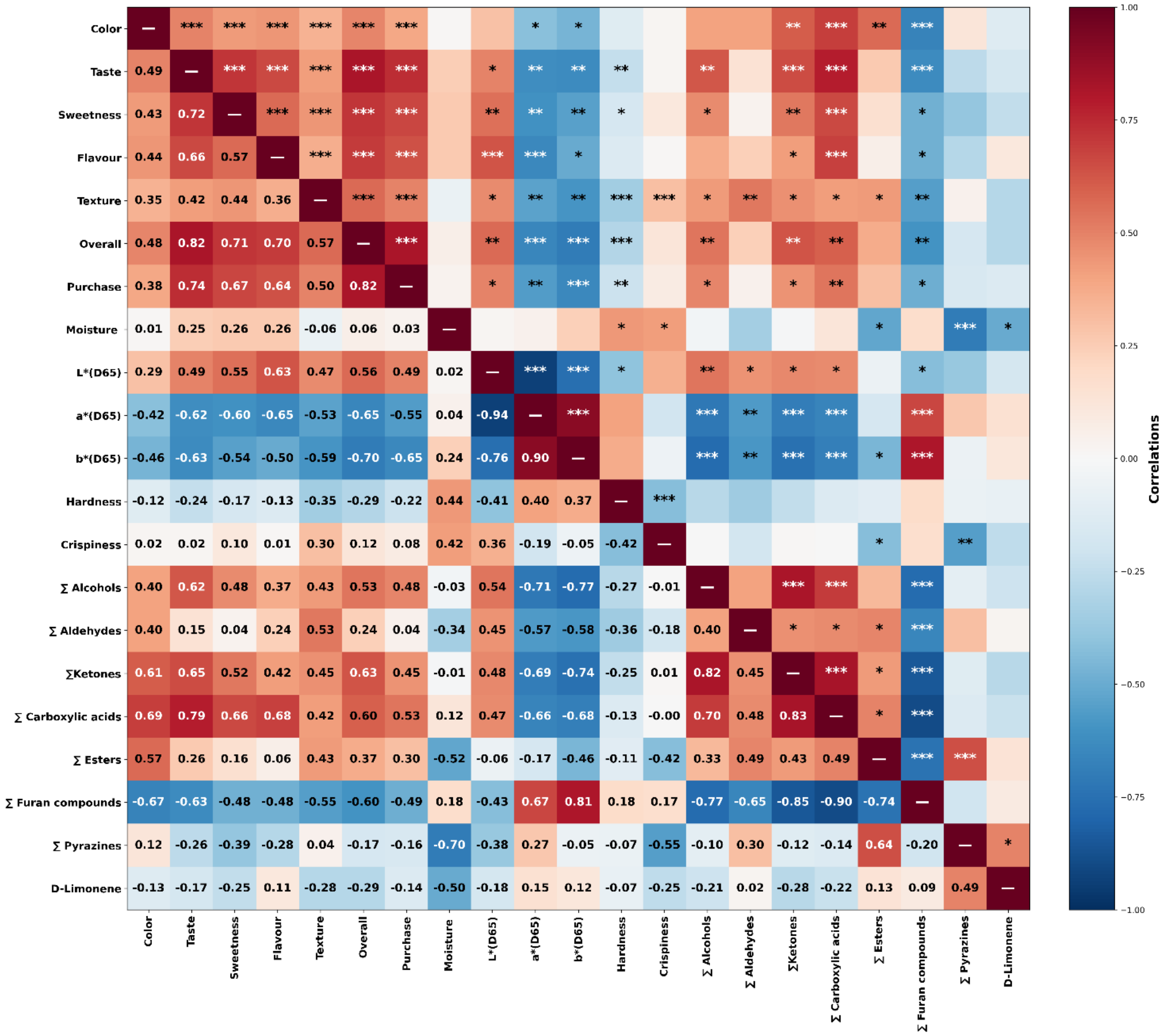

Figure S4. Pearson correlation matrix between the identified volatile compounds (expressed as sums) and the sensory analysis scores of the biscuits

Colour intensity indicates the strength of the correlation. The statical significance of the correlation is reported in the upper/right part of the figure (where * = p < 0.05; ** = p<0.01; *** = p<0.001).
