# Supplementary material for: Impact of Sucrose Replacement on Physicochemical Properties of Whole-Wheat Biscuits
Source: Foods. 2026 Jun 5;15(11):2032. doi: 10.3390/foods15112032 (PMC13256550; doi:10.3390/foods15112032)
Supplement: Supplementary file 1 [file foods-15-02032-s001.zip › Table S1.pdf]

Table S1. Volatile compounds detected in biscuits produced with sucrose and different replacers.

**Volatile compounds (%)**	**Erythritol**	**Isomalt**	**Maltitol**	**Sorbitol**	**Xylitol**	**Fructose**	**Trehalose**	**Sucrose**	***Sig.***
**Alcohols**									
Ethanol	2.23± 0.08^{c}	2.34± 0.03^{bc}	2.51± 0.10^{abc}	2.77± 0.64^{ab}	2.75± 0.28^{ab}	1.21± 0.07^{d}	2.87± 0.26^{a}	2.80 ± 0.02^{ab}	***
1-Pentanol	n.d.^{d}	n.d.^{d}	n.d.^{d}	n.d.^{d}	0.27± 0.00^{a}	0.13± 0.00^{c}	0.21± 0.01^{b}	0.25 ± 0.07^{ab}	***
1-Hexanol	0.34± 0.02^{bc}	0.42± 0.12^{ab}	0.44± 0.03^{a}	0.32± 0.01^{cd}	0.35± 0.02^{abc}	0.23± 0.01^{d}	0.32± 0.00^{cd}	0.30 ± 0.06^{cd}	**
Hexanol	n.d.^{c}	n.d.^{c}	n.d.^{c}	1.46± 0.12^{a}	n.d.^{c}	n.d.^{c}	0.82± 0.04^{b}	0.89 ± 0.08^{b}	***
Heptanol	0.87± 0.11^{abc}	0.89± 0.05^{ab}	0.97± 0.08^{a}	0.89± 0.03^{ab}	0.65± 0.07^{de}	0.57± 0.04^{e}	0.79± 0.09^{bc}	0.74 ± 0.07^{cd}	***
1-Octanol	1.32± 0.26^{a}	1.33± 0.05^{a}	1.35± 0.19^{a}	1.11± 0.16^{ab}	1.24± 0.03^{ab}	1.04± 0.08^{b}	1.01± 0.03^{b}	1.02 ± 0.15^{b}	*
1-Octen-3-ol	0.76± 0.01^{b}	0.77± 0.05^{b}	0.94± 0.00^{a}	0.79± 0.02^{b}	0.67± 0.02^{c}	0.48± 0.01^{d}	0.77± 0.11^{b}	0.78 ± 0.01^{b}	***
Benzyl alcohol	1.32± 0.27	1.20± 0.71	1.39± 0.18	1.23± 0.08	1.39± 0.31	1.08± 0.12	1.12± 0.20	1.13 ± 0.23	n.s
**∑ Alcohols**	**6.84 ± 0.52^{c}**	**6.95 ± 0.78^{c}**	**7.60 ± 0.28^{bc}**	**8.57 ± 0.72^{a}**	**7.32 ± 0.10^{bc}**	**4.74 ± 0.07^{d}**	**7.91 ± 0.33^{ab}**	**7.91 ± 0.46^{ab}**	***
**Aldehydes**									
2-Methylbutanal	4.99± 0.32^{a}	4.88± 0.04^{a}	4.96± 0.13^{a}	4.21± 0.00^{b}	4.98± 0.81^{b}	4.00± 0.01^{b}	4.10± 0.30^{b}	4.07± 0.40^{b}	**
3-Methylbutanal	4.37± 0.24	4.45± 0.12	4.42± 0.26	4.43± 0.12	4.32± 0.42	4.10± 0.08	4.50± 0.01	4.47± 0.46	n.s
Pentanal	3.70± 0.09^{bc}	3.96± 0.11^{a}	3.78± 0.04^{b}	3.51± 0.10^{d}	3.60± 0.02^{cd}	2.07± 0.05^{f}	2.90± 0.18^{e}	2.89± 0.06^{e}	***
2-pentanal	n.d.^{c}	1.56± 0.13^{ab}	n.d.^{c}	1.72± 0.33^{a}	n.d.^{c}	1.33± 0.07^{b}	1.53± 0.04^{ab}	1.44± 0.06^{b}	***
Hexanal	4.83± 0.17^{ab}	4.67± 0.02^{bc}	4.99± 0.14^{a}	4.45± 0.16^{cde}	4.39± 0.21^{de}	4.22± 0.02^{e}	4.34± 0.00^{e}	4.58± 0.05^{cd}	***
Heptanal	1.52± 0.18^{ab}	1.88± 0.06^{a}	1.86± 0.05^{a}	1.32± 0.01^{b}	1.34± 0.44^{b}	1.72± 0.02^{a}	1.80± 0.02^{a}	1.67± 0.27^{ab}	*
Nonanal	4.37± 0.25^{c}	4.93± 0.03^{a}	4.72± 0.02^{b}	4.29± 0.13^{cd}	4.28± 0.05^{cd}	4.13± 0.02^{d}	4.22± 0.07^{cd}	4.33± 0.17^{cd}	***
(E,E)-2,4-Decadienal	2.57± 0.15	3.00± 0.04	2.77± 0.01	2.76± 0.14	2.67± 0.41	2.72± 0.06	3.00± 0.14	2.89± 0.21	n.s
Benzaldehyde	1.29± 0.12b^{c}	1.53± 0.16^{ab}	1.56± 0.06^{a}	1.38± 0.27^{ab}	1.00± 0.13^{de}	0.69± 0.07^{f}	1.09± 0.16^{cd}	0.79± 0.01^{ef}	***
Phenylacetaldehyde	n.d.^{c}	n.d.^{c}	n.d.^{c}	n.d.^{c}	0.12± 0.00^{b}	n.d.^{c}	n.d.^{c}	0.24± 0.01^{a}	***
**∑ Aldehydes**	**27.64 ± 1.34^{bc}**	**30.86 ± 0.08^{a}**	**29.06 ± 0.35^{b}**	**28.07 ± 0.67^{bc}**	**26.70 ± 1.78^{c}**	**24.98 ± 0.27^{d}**	**27.48 ± 0.68^{c}**	**27.37 ± 1.10^{c}**	***
**Ketones**									
Acetone	8.45± 0.65^{a}	8.57± 0.45^{a}	8.56± 0.78^{a}	8.56± 0.67^{a}	8.45± 0.34^{a}	3.34± 0.04^{c}	4.32± 0.08^{b}	4.23± 0.06^{b}	***
3-Hydroxy-2- Butanone (Acetoin)	1.99± 0.41	1.88± 0.33	1.98± 0.62	2.26± 0.45	1.90± 0.60	2.08± 0.04	2.65± 0.63	2.72± 0.44	n.s
2-Pentanone	2.34± 0.22	2.76± 0.21	2.45± 0.82	2.65± 0.03	2.82± 0.03	2.66± 0.11	2.73± 0.45	2.74± 0.07	n.s
4-cyclopentene-1,3 dione	1.78± 0.08^{a}	1.26± 0.06^{b}	1.34± 0.23^{b}	1.92± 0.21^{a}	1.95± 0.05^{a}	1.24± 0.12b^{c}	1.01± 0.04^{c}	1.01± 0.07^{c}	***
2-Heptanone	8.76± 0.09^{a}	9.35± 0.06^{a}	8.99± 0.12^{a}	8.99± 0.13^{a}	8.78± 0.08^{a}	6.78± 0.89^{b}	8.98± 0.42^{a}	8.76± 1.00^{a}	***
2-Nonanone	4.34± 0.01^{a}	4.63± 0.08^{a}	4.66± 0.03^{a}	4.58± 0.90^{a}	4.24± 0.04^{a}	3.25± 0.08^{b}	4.09± 0.00^{a}	4.10± 0.87^{a}	*
2-Undecanone	0.88± 0.01^{ab}	0.87± 0.09^{ab}	0.86± 0.09^{b}	0.99± 0.01^{a}	0.78± 0.13^{bc}	n.d.^{d}	0.71± 0.06^{c}	0.66± 0.03^{c}	***
4-Hydroxy-4-methyl-2-pentanone	1.33± 0.01^{cd}	0.98± 0.10^{d}	1.00± 0.18^{d}	1.29± 0.02^{cd}	1.56± 0.25^{c}	4.89± 0.02^{b}	6.72± 0.06^{a}	6.80± 0.47^{a}	***

**∑ Ketones**	**29.87 ± 0.75^{a}**	**30.30 ± 0.46^{a}**	**29.84 ± 1.05^{a}**	**31.24 ± 1.95^{a}**	**30.48 ± 0.67^{a}**	**24.24 ± 0.98^{b}**	**31.21 ± 0.79^{a}**	**31.02 ± 1.26^{a}**	***
**Carboxylic acids**									
Acetic acid	4.54± 0.29^{a}	4.53± 0.86^{a}	4.44± 0.03^{a}	4.46± 0.38^{a}	4.45± 0.45^{a}	2.31± 0.19^{b}	4.43± 0.02^{a}	4.62± 0.58^{a}	***
Propanoic acid	2.81± 0.64^{a}	2.91± 0.12^{a}	2.91± 0.06^{a}	2.88± 0.60^{a}	1.98± 0.17^{b}	n.d.^{c}	2.23± 0.42^{ab}	2.23± 0.35^{ab}	***
Butanoic acid	0.34± 0.03^{d}	0.45± 0.02^{cd}	n.d.^{e}	0.67± 0.07^{b}	0.56± 0.03^{bc}	n.d.^{e}	1.01± 0.24^{a}	1.03± 0.08^{a}	***
Hexanoic acid	1.45± 0.02^{ab}	1.51± 0.02^{a}	1.56± 0.05^{a}	1.35± 0.18^{abc}	1.38± 0.17^{abc}	0.91± 0.02^{d}	1.23± 0.20^{bc}	1.20± 0.09^{c}	***
Octanoic acid	1.98± 0.34^{a}	n.d.^{b}	n.d.^{b}	n.d.^{b}	2.45± 0.16^{a}	n.d.^{b}	2.23± 0.80^{a}	1.96± 0.63^{a}	***
2-Hexenoic acid	1.21± 0.19^{a}	1.34± 0.02^{a}	1.30± 0.03^{a}	1.20± 0.13^{a}	1.02± 0.10^{b}	0.89± 0.03^{b}	0.97± 0.03^{b}	0.98± 0.06^{b}	***
Nonanoic acid	2.30± 0.25^{a}	2.31± 0.01^{a}	2.38± 0.24^{a}	2.12± 0.00^{ab}	2.23± 0.12^{ab}	1.45± 0.12^{c}	2.00± 0.10^{b}	2.02± 0.08^{b}	***
Decanoic acid	1.98± 0.22	1.97± 0.04	2.03± 0.37	2.00± 0.08	2.01± 0.39	1.76± 0.01	2.17± 0.01	2.21± 0.08	n.s.
**∑ Carboxylic acids**	**16.61± 0.59^{a}**	**15.02± 0.81^{bc}**	**14.62± 0.13^{c}**	**14.68± 0.78^{c}**	**16.08± 0.68^{ab}**	**7.32± 0.32^{d}**	**16.27± 0.98^{ab}**	**16.25± 0.99^{ab}**	***
**Esters**									
Ethyl acetate	2.84± 0.01^{b}	2.81± 0.02^{b}	2.85± 0.28^{b}	2.34± 0.24^{b}	4.23± 1.23^{a}	n.d.^{c}	0.12± 0.00^{c}	n.d.^{c}	***
Ethyl butyrate	2.58± 0.05^{a}	2.67± 0.04^{a}	2.55± 0.38^{a}	2.78± 0.14^{a}	2.67± 0.98^{a}	0.78± 0.01^{b}	0.75± 0.02^{b}	n.d.^{c}	***
Butyl acetate	0.78± 0.02^{b}	0.76± 0.03^{b}	0.87± 0.09^{a}	0.45± 0.08^{d}	0.28± 0.01^{e}	0.39± 0.01^{d}	0.67± 0.01^{c}	n.d.^{f}	***
Butyl 3-methylbutyrate	0.56± 0.19^{a}	0.54± 0.01^{a}	0.51± 0.01^{a}	0.56± 0.03^{a}	0.25± 0.01^{b}	0.49± 0.02^{a}	0.60± 0.01^{a}	n.d.^{c}	***
Ethyl hexanoate	n.d.^{c}	n.d.^{c}	n.d.^{c}	n.d.^{c}	0.10± 0.00^{b}	n.d.^{c}	n.d.^{c}	1.82± 0.06^{a}	***
Methyl benzoate	n.d.^{c}	n.d.^{c}	n.d.^{c}	n.d.^{c}	0.23± 0.02^{b}	n.d.^{c}	n.d.^{c}	1.61± 0.02^{a}	***
Ethyl benzoate	n.d.^{c}	n.d.^{c}	n.d.^{c}	n.d.^{c}	0.32± 0.02^{a}	n.d.^{c}	n.d.^{c}	0.25± 0.00^{b}	***
**∑ Esters**	**6.76± 0.24^{b}**	**6.78± 0.04^{b}**	**6.78± 0.48^{b}**	**6.13± 0.33^{c}**	**8.08± 0.28^{a}**	**1.66± 0.03^{f}**	**2.14± 0.02^{e}**	**3.68± 0.04^{d}**	***
**Furan compounds**									
2-Pentylfuran	1.59± 0.35	1.56± 0.17	1.52± 0.01	1.40± 0.02	1.34± 0.01	1.58± 0.01	1.52± 0.04	1.56± 0.02	n.s.
2-Furanmethanol	1.98± 0.03^{c}	1.97± 0.06^{c}	1.96± 0.51^{c}	1.36± 0.06^{d}	1.89± 0.43^{c}	3.45± 0.03^{a}	2.77± 0.08^{b}	2.66± 0.09^{b}	***
Furfural	1.17± 1.18^{e}	1.34± 0.02^{cd}	1.12± 0.20^{e}	1.10± 0.12^{e}	1.22± 0.07^{cd}	15.35± 0.03^{a}	2.33± 0.45^{b}	2.04± 0.18^{bc}	***
Furaneol	0.56± 0.00^{d}	0.22± 0.01^{e}	0.55± 0.01^{d}	0.56± 0.02^{d}	0.45± 0.00^{d}	3.24± 0.08^{a}	1.67± 0.17^{c}	1.79± 0.04^{b}	***
5-Hydroxymethylfurfural (HMF)	1.77± 0.04^{c}	1.12± 0.06^{d}	1.75± 0.26^{c}	1.74± 0.14^{c}	1.68± 0.02^{c}	4.23± 0.54^{a}	2.45± 0.10^{b}	1.95± 0.25^{c}	***
Furfuryl alcohol	2.31± 0.05^{b}	2.20± 0.21^{b}	2.27± 0.22^{b}	2.31± 0.27^{b}	2.33± 0.02^{b}	6.67± 0.83^{a}	2.45± 0.02^{b}	2.43± 0.13^{b}	***
**∑ Furan compounds**	**9.38± 1.24^{c}**	**8.41± 0.42^{c}**	**9.17± 0.94^{c}**	**8.47± 0.39^{c}**	**8.91± 0.48^{c}**	**34.52± 1.09^{a}**	**13.19± 0.32^{b}**	**12.43± 0.32^{b}**	***
**Pyrazines**									
2,5-Dimethylpyrazine	0.64± 0.01^{a}	0.66± 0.00^{a}	0.62± 0.00^{ab}	0.65± 0.09^{a}	0.58± 0.00^{b}	0.67± 0.02 ^{a}	0.34± 0.01^{c}	n.d.^{d}	***
Methylpyrazine	0.41± 0.01^{ab}	0.38± 0.02^{b}	0.45± 0.01^{a}	0.39± 0.08^{b}	0.23± 0.01^{c}	0.20± 0.02^{c}	n.d.^{d}	0.21± 0.01^{c}	***
2-Methylpyrazine	0.28± 0.00^{b}	0.22± 0.01^{c}	0.29± 0.00^{b}	0.45± 0.05^{a}	0.20± 0.02^{c}	0.12± 0.00^{d}	n.d.^{e}	n.d.^{e}	***
**∑ Pyrazines**	**1.33± 0.01^{b}**	**1.26± 0.03^{c}**	**1.36± 0.01^{b}**	**1.49± 0.08^{a}**	**1.01± 0.01^{d}**	**0.99± 0.02^{d}**	**0.34± 0.01^{e}**	**0.21± 0.01^{f}**	***

**Terpenes**<br>D-Limonene	1.54± 0.32	1.53± 0.01	1.56± 0.08	1.45± 0.10	1.45± 0.22	1.55± 0.03	1.45± 0.02	1.11± 0.03	**n.s**

Results of ANOVA with Duncans's post hoc test are reported between samples; values followed by different letters are significantly different at $p < 0.05$. Sig., statistical significance; n.s., not significant; the asterisks denote the level of significance: *$p<0.05$, **$p<0.01$, ***$p < 0.001$.
