# Supplementary material for: Impact of Sucrose Replacement on Physicochemical Properties of Whole-Wheat Biscuits
Source: Foods. 2026 Jun 5;15(11):2032. doi: 10.3390/foods15112032 (PMC13256550; doi:10.3390/foods15112032)
Supplement: Supplementary file 1 [file foods-15-02032-s001.zip › Table S2.pdf]

Table S2: Average values obtained for the biscuits during sensory analysis. Results of colour, taste, sweetness, flavour, texture and overall liking are reported as score out to 9 (9-point hedonistic scale), whereas those of purchase predisposition as score out to 7 (7-point hedonistic scale).

	**Erythritol**	**Isomalt**	**Maltitol**	**Sorbitol**	**Xylitol**	**Fructose**	**Trehalose**	**Sucrose**
**Colour**	5.90 ± 2.07	6.70 ± 1.16	6.30 ± 1.67	6.45 ±1.74	6.05 ± 1.34	3.60 ± 2.16	5.85 ± 2.23	6.30 ± 1.51
**Taste**	4.00 ± 1.69	5.55 ± 1.34	4.95 ± 1.55	4.25 ± 1.19	5.85 ± 1.17	2.00 ± 0.96	5.30 ± 2.08	6.00 ± 1.20
**Sweetness**	4.00 ± 1.81	4.50 ± 1.88	4.45 ± 2.04	4.05 ± 1.68	5.75 ± 1.46	2.60 ± 1.77	5.25 ± 2.44	5.55 ± 2.21
**Flavour**	5.25 ± 1.75	5.65 ± 1.51	4.85 ± 1.67	3.93 ± 1.23	5.05 ± 1.52	2.60 ± 1.91	5.70 ± 1.95	5.50 ± 1.62
**Texture**	3.40 ± 1.88	6.50 ± 1.38	6.43 ± 1.63	5.95 ± 1.71	5.95 ± 1.08	5.15 ± 2.65	6.50 ± 2.11	6.15 ± 1.87
**Overall**	4.00 ± 1.50	5.75 ± 1.15	5.05 ± 1.48	4.45 ± 0.93	5.80 ± 1.49	2.55 ± 1.80	5.35 ± 1.90	6.20 ± 1.49
**Purchase Interest**	2.80 ± 1.26	3.80 ± 1.62	3.40 ± 1.58	2.95 ± 1.08	4.50 ± 0.93	1.70 ± 1.24	3.80 ± 1.74	4.05 ± 1.26
