# Supplementary figures and images for: Impact of Sucrose Replacement on Physicochemical Properties of Whole-Wheat Biscuits
Source: Foods. 2026 Jun 5;15(11):2032. doi: 10.3390/foods15112032 (PMC13256550; doi:10.3390/foods15112032)

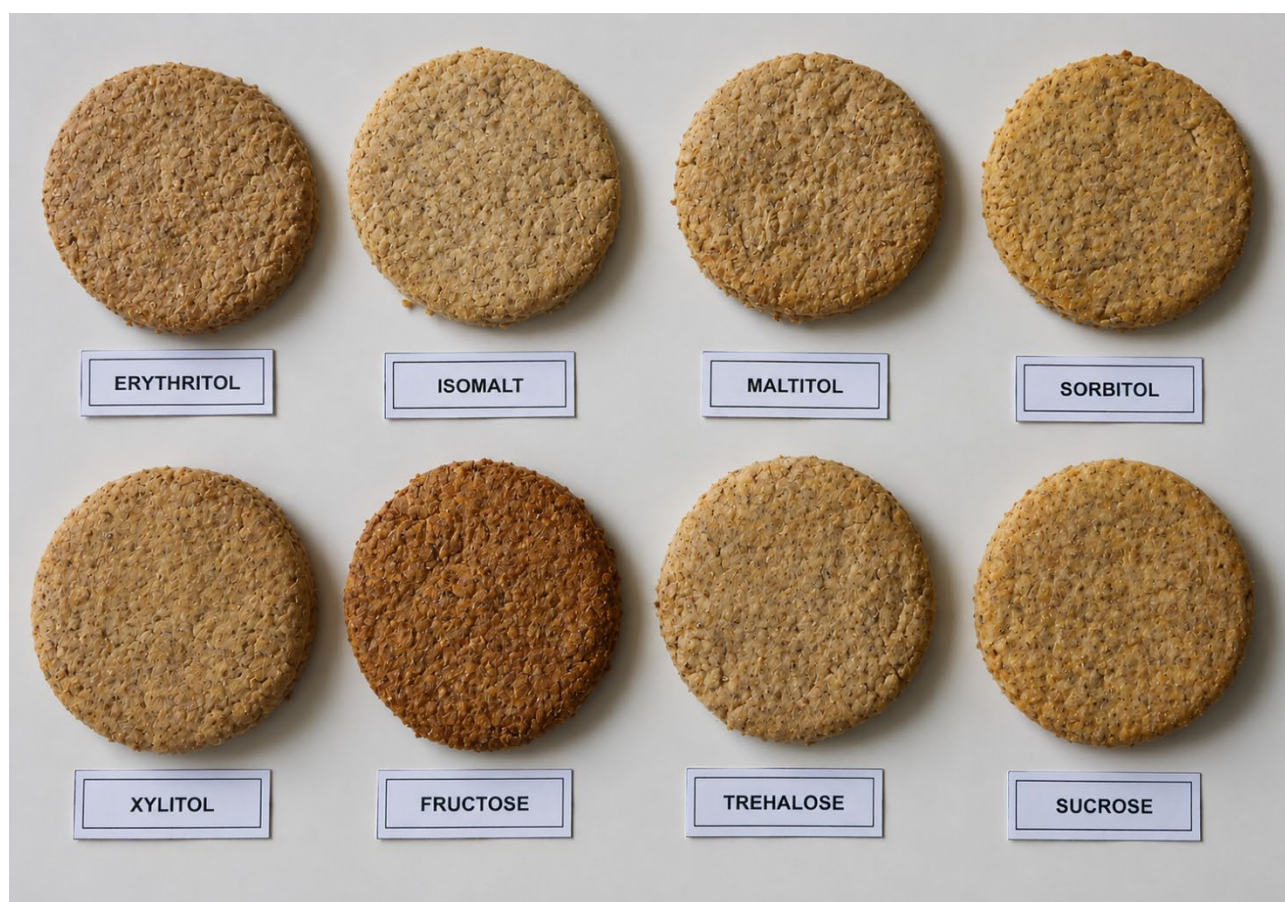

**Figure S2.** Visual comparison between the different biscuits.

Supplement: Supplementary file 1 [file foods-15-02032-s001.zip › Figure S2.pdf]
